# BRIDGE: Burr hole vs. craniotomy - radiomic indicators determining the chronification grid in acute subdural hematoma evaluation

**DOI:** 10.1016/j.bas.2026.106034

**Published:** 2026-04-06

**Authors:** Alexandru Guranda, Johannes Wach, Sebastian Lehmann, Felix Arlt, Alim Emre Basaran, Tim Wende, Erdem Güresir, Martin Vychopen

**Affiliations:** Department of Neurosurgery, University Hospital Leipzig, 04103, Leipzig, Germany

**Keywords:** Acute subdural hematoma, Burr-hole surgery, Hematoma chronification, Radiomic, Surgical decision-making, Craniotomy

## Abstract

**Introduction:**

Acute subdural hematoma (aSDH) is commonly treated by craniotomy. In some patients, however, the hematoma evolves into a chronic subdural hematoma (cSDH), which can be safely evacuated by delayed burr-hole trepanation — a less invasive procedure associated with lower morbidity.

**Research question:**

This study aimed to identify clinical, laboratory, and radiomic predictors of hematoma chronification requiring delayed burr-hole trepanation in aSDH.

**Material and methods:**

After excluding patients with decompressive hemicraniectomy, those with contusions, and patients with epidural hematoma, we retrospectively analyzed 118 patients with traumatic aSDH admitted between 2015 and 2022. Patients underwent either primary craniotomy (n = 64), developed chronification requiring burr-hole trepanation (n = 16), or were managed conservatively without subsequent surgery (n = 38). Clinical, laboratory, and radiomics-derived variables were assessed.

**Results:**

The burr-hole group had lower SOFA scores (p = 0.034), lower leukocyte counts (p = 0.043), and fewer postoperative seizures (13% vs. 44%, p = 0.023). Radiomic analysis revealed significantly higher hematoma elongation (p = 0.001), which remained the sole independent predictor of chronification (OR 22.0, 95% CI 3.7–132.6; p = 0.001). Compared with conservative management, the burr-hole group was more frequently male (p = 0.049), had lower leukocyte counts (p = 0.031), and showed higher elongation (p = 0.008) and sphericity (p = 0.004). In multivariable analysis, elongation independently predicted chronification (OR 4.0, 95% CI 1.2–13.1; p = 0.021).

**Conclusion:**

Radiomic parameters support the stratification of patients who are more likely to benefit from delayed burr-hole trepanation rather than acute craniotomy.

## Highlights

• Hematoma shape predicts delayed burr-hole surgery after acute subdural hematoma.
• Elongated hematomas are more likely to progress to chronic subdural hematoma.
• Imaging-based shape analysis outperforms hematoma size for risk prediction.
• Selected patients may safely avoid emergency craniotomy.

## 1 Introduction

The standard of care for aSDH with mass effect is surgical evacuation with two surgical options: craniotomy or decompressive craniectomy. Both procedures represent a relatively invasive therapy, which might cause additional complications (Hutchinson et al., 2023; Solomou et al., 2024; Trevisi et al., 2020).

In contrast, a subset of aSDH patients does not necessitate acute evacuation, as their hematoma gradually evolves into a cSDH that can be effectively treated with minimally invasive burr-hole trepanation. This approach is regarded as the first-line surgical strategy for cSDH, providing the most favorable balance between efficacy and complication risk (Solou et al., 2022). From a patient-centered perspective, identifying individuals who may safely undergo delayed burr-hole evacuation instead of emergency craniotomy could represent a paradigm shift in aSDH management.

However, reliable predictors for this “chronification pathway” remain unclearly defined. Although factors such as hematoma size and midline shift have repeatedly been suggested, their prognostic value has proven inconsistent across studies (Liebert et al., 2025). Clinical parameters such as age and comorbidities are relevant for short-term prognosis, but they are insufficient to guide long-term management decisions (Sharma et al., 2020). Thus, a precise risk stratification tool distinguishing patients who require immediate craniotomy from those whose aSDH can remain untreated and is likely to undergo chronification is needed.

In recent years, radiomics has enabled the extraction of quantitative imaging parameters describing geometry, morphology, and texture beyond visual assessment, providing novel tools for outcome prediction. In oncology and stroke, radiomics has already shown predictive value and contributed to treatment planning (Liu et al., 2019; Chen et al., 2021). Its application in traumatic subdural hematoma, however, remains in its infancy.

Our research group has systematically explored radiomics and clinical predictors in aSDH across a series of studies. The RADAR study demonstrated that hematoma surface area, rather than volume, predicts functional outcome and survival after craniotomy (Richter et al., 2025). The PROMISE study extended this analysis to postoperative imaging, identifying Δ surface area and preoperative Feret diameter as independent predictors of 30-day outcome (Guranda et al., 2025a). Complementing these radiomic approaches, the KEPPRA study focused on epilepsy as a clinical prognostic factor, revealing its strong association with unfavorable neurological recovery (Guranda et al., 2025b). Together, these projects established a strong foundation for integrating radiomic and clinical data into aSDH prognostication.

The present BRIDGE study builds upon this line of research by addressing the challenge of predicting hematoma chronification suitable for safe burr-hole evacuation in aSDH. The study specifically aimed to identify clinical, laboratory, and radiomic predictors associated with delayed burr-hole trepanation following aSDH. By integrating these parameters, this study seeks to bridge acute and chronic subdural hematoma management and provide neurosurgeons with tools to support individualized decision-making, thereby potentially sparing selected patients from unnecessary high-risk craniotomies.

## 2 Methods

This retrospective single-center cohort study was conducted at the Department of Neurosurgery, University Hospital Leipzig, in accordance with the Declaration of Helsinki. Ethical approval was obtained from the institutional review board (protocol number 362/23-ek).

All consecutive adult patients (≥18 years) admitted with traumatic aSDH between January 2015 and December 2022 were screened for eligibility. Inclusion criteria were: (i) CT-confirmed aSDH on admission, (ii) availability of diagnostic cranial CT within 24 h after trauma, and (iii) complete clinical documentation during index hospitalization. Exclusion criteria were: (i) primarily chronic subdural hematoma, additional intracranial pathology (e.g. contusion, epidural hematoma), (ii) prior intracranial surgery during the same episode, and (iii) non-diagnostic or missing CT data (iv) patients who underwent decompressive craniectomy.

For the purpose of analysis, patients were allocated to three mutually exclusive treatment groups. The first group comprised individuals who underwent primary craniotomy (n = 64), defined as urgent surgical evacuation during the admission. The second group included patients who initially received non-operative management but subsequently developed hematoma chronification requiring burr-hole trepanation (n = 16). Successful chronification was determined on a follow-up CT scan performed 7–10 days after the initial injury, based on multidisciplinary consensus between the attending neurosurgeon and neuroradiologist. The third group consisted of patients managed conservatively (n = 38), who did not undergo surgical intervention during the initial hospitalization or follow-up.

Non-contrast cranial CT was performed in accordance with institutional trauma protocols. Standard acquisition comprised 1-mm axial sections. Admission CT served as the basis for all radiomic analyses, while follow-up scans were obtained as clinically indicated. All images were exported in DICOM format for subsequent evaluation.

Radiomic segmentation and analysis were conducted using 3D Slicer (version 5.2.2). Hematomas were segmented with a semi-automated grow-from-seeds workflow, followed by manual refinement. Two neurosurgeons (A.G. and M.V.), blinded to patient outcomes, independently reviewed all segmentations, and discrepancies were resolved by JW. Extracted radiomic features included hematoma volume (cm^3^), surface area (mm^2^), Feret diameter (mm), elongation, flatness, roundness, and sphericity. All radiomic measurements were derived exclusively from the admission CT scans to ensure a uniform baseline across treatment groups.

**Fig. 1:**
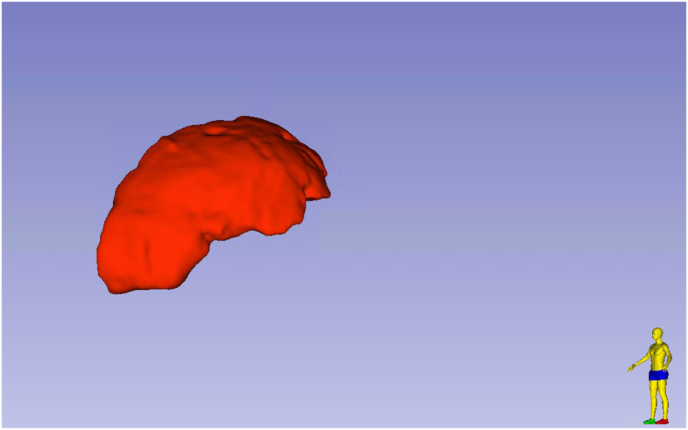
Representative three-dimensional segmentation of acute subdural hematoma based on 1-mm axial admission CT. Shape descriptors (elongation, flatness, roundness, and sphericity) were calculated from the volumetric binary labelmap using 3D Slicer, reflecting true three-dimensional morphology.

Shape descriptors were derived from the binary three-dimensional labelmap representation of the segmented hematoma using the Segment Statistics module in 3D Slicer, based on the standardized implementation described in the official software documentation (www.slicer.org). Elongation and flatness were calculated from the principal moments of inertia of the voxel distribution, reflecting deviation from spherical symmetry along major and minor axes. Roundness was defined as the ratio between the surface area of a sphere derived from the Feret diameter and the actual surface area of the segmented volume (value of 1 representing a perfect sphere). Sphericity was computed based on the surface-area–to–volume relationship and quantifies similarity to an ideal sphere. A representative three-dimensional segmentation example is provided in Fig. 1.

The following variables were retrieved from electronic records: age, sex, admission GCS, pupillary status at admission (isocoric vs. anisocoric), history of antithrombotic therapy, arterial hypertension, cardiac comorbidities (coronary artery disease, prior myocardial infarction, heart failure, arrhythmias, valvular disease, cardiomyopathies, prior cardiac surgery), postoperative SOFA score, postoperative APACHE score, and occurrence of epileptic seizures after treatment. Laboratory markers included leukocyte count, hematocrit, and hemoglobin on admission.

For seizure assessment, both focal and generalized seizures were combined into a single binary variable (“postoperative epilepsy”). Postoperative epilepsy was defined as any epileptic event occurring during hospitalization after the index trauma and/or surgical intervention, which was confirmed by electroencephalography (EEG), and which prompted initiation of antiepileptic medication.

The primary endpoint was hematoma chronification requiring delayed burr-hole trepanation. Analyses were conducted in two comparisons: (i) patients with chronification versus those undergoing primary craniotomy, and (ii) patients with chronification versus those managed conservatively. The aim of the study was to identify clinical, laboratory, and radiomic indicators associated with this chronification pathway.

## 3 Statistics

Continuous variables were tested for normality using the Shapiro–Wilk test and are presented as mean ± standard deviation (SD) or median with interquartile range (IQR), as appropriate. Categorical variables are reported as absolute numbers and percentages. Between-group comparisons employed Student's t-test for continuous variables and Chi-squared test or Fisher's exact test for categorical variables. A two-tailed p < 0.05 was considered statistically significant.

Variables with *p* < 0.05 in univariable analysis were entered into multivariable binary logistic regression. To reduce overfitting in the small chronification group (n = 16), the number of predictors was restricted to maintain an events-per-variable ratio ≥10. Multicollinearity was assessed using Spearman correlation and variance inflation factor (VIF), with no relevant collinearity detected. Logistic regression was performed with backward stepwise likelihood ratio selection in SPSS (version 29, IBM, Armonk, NY, USA).

Additional sensitivity analyses were performed in R (version 4.4.2; R Foundation for Statistical Computing, Vienna, Austria). Standard logistic regression was conducted using the glm() function (stats package). Penalized logistic regression was performed using the logistf package (Firth correction). Bootstrap validation (1000 resamples) was implemented using the boot package, and leave-one-out sensitivity analysis was conducted to assess the influence of individual observations.

## 4 Results

### 4.1 Patient characteristics

A total of 118 patients with aSDH met inclusion criteria. The cohort consisted of 64 (54.2%) patients undergoing primary craniotomy, 16 (13.6%) patients developing chronification with delayed burr-hole trepanation, and 38 (32.2%) patients managed conservatively. The median age was 77 years [IQR 20], and 57% were male.

Baseline clinical, laboratory, and therapeutic characteristics of patients treated with delayed burr-hole trepanation and those undergoing primary craniotomy were compared. The results of the univariable analysis are presented in Table 1.

**Table 1:** Univariate analysis comparing delayed burr-hole trepanation and primary craniotomy.

Variable	Craniotomy (n = 64)	Burr-hole (n = 16)	*p*-Value
***Demographics***			
Age, years median [IQR]	77.0 [20]	80.5 [19]	0.056
Male sex, n (%)	36 (56%)	11 (69%)	0.364
***Clinical presentation***			
Pupillary abnormality, n (%)	13 (20%)	1 (6%)	0.280
SOFA score, median (IQR)	3.5 [4]	2 [2]	0.034
APACHE score, median (IQR)	15 [9]	12 [5]	0.152
***Comorbidities/Therapy***			
Cardiac comorbidity, n (%)	45 (70%)	11 (69%)	1.000
Anticoagulation, n (%)	33 (52%)	10 (63%)	0.433
***Laboratory values***			
Hemoglobin, mmol/L (mean ± SD)	7.34 ± 1.21	7.80 ± 1.20	0.179
Hematocrit, L/L (mean ± SD)	0.34 ± 0.05	0.36 ± 0.05	0.100
Thrombocytes, G/L (mean ± SD)	223.26 ± 244.81	218.13 ± 64.51	0.934
Leukocytes, G/L (mean ± SD)	10.99 ± 4.89	8.30 ± 3.65	0.043
Quick, % (mean ± SD)	85.81 ± 19.12	86.13 ± 30.97	0.959
aPTT, sec (mean ± SD)	27.15 ± 4.19	29.70 ± 8.30	0.086
***Outcome***			
Epilepsy, n (%)	28 (44%)	2 (13%)	0.023

Patients in the burr-hole group had significantly lower SOFA scores (*p* = 0.034) and lower leukocyte counts (*p* = 0.043) compared with those undergoing craniotomy. Moreover, postoperative epileptic seizures occurred markedly less often in the burr-hole group (*p* = 0.023). No other variables differed significantly between groups.

To further investigate predictors of hematoma chronification, patients who required delayed burr-hole trepanation were compared with those managed conservatively without surgery. The results of the univariable analysis are presented in Table 2.

**Table 2:** Univariate analysis comparing delayed burr-hole trepanation and conservative.

Variable	Burr-hole (n = 16)	Conservative (n = 38)	*p*-Value
***Demographics***			
Age, years median [IQR]	80.5 [19]	83.0 [18]	0.393
Male sex, n (%)	11 (69%)	15 (39.5%)	0.049
***Clinical presentation***			
Pupillary abnormality, n (%)	1 (6%)	5 (13.2%)	0.657
SOFA score, median (IQR)	2 [2]	3 [5]	0.216
APACHE score, median (IQR)	12 [5]	12 [9]	0.796
***Comorbidities/Therapy***			
Cardiac comorbidity, n (%)	11 (69%)	30 (78.9%)	0.493
Anticoagulation, n (%)	10 (63%)	19 (50%)	0.400
***Laboratory values***			
Hemoglobin, mmol/L (mean ± SD)	7.80 ± 1.20	7.82 ± 0.97	0.946
Hematocrit, L/L (mean ± SD)	0.36 ± 0.05	0.36 ± 0.04	0.927
Thrombocytes, G/L (mean ± SD)	218.13 ± 64.51	210.87 ± 75.20	0.738
Leukocytes, G/L (mean ± SD)	8.30 ± 3.65	10.80 ± 3.85	0.031
Quick, % (mean ± SD)	86.13 ± 30.97	81.58 ± 25.37	0.576
aPTT, sec (mean ± SD)	29.70 ± 8.30	27.49 ± 6.26	0.288
***Outcome***			
Epilepsy, n (%)	2 (13%)	6 (15.8%)	1.000

In univariable analysis, patients in the burr-hole group were more frequently male compared with those managed conservatively (*p* = 0.049). Leukocyte counts were also significantly lower in the burr-hole group (*p* = 0.031). To further address our primary objective, we evaluated radiomic parameters between treatment groups. Table 3 summarizes the comparison of delayed burr-hole trepanation and primary craniotomy, while Table 4 presents the comparison of delayed burr-hole trepanation and conservative management.

**Table 3:** Univariate analysis of radiomics comparing delayed burr-hole trepanation and primary craniotomy.

Variable	Craniotomy (n = 64)	Burr-hole (n = 16)	*p*-Value
Elongation	1.53 ± 0.29	2.03 ± 0.65	0.001
Feret Diameter (mm)	144.83 ± 24.93	137.57 ± 29.91	0.320
Sphericity	0.27 ± 0.89	0.34 ± 0.15	0.756
Surface area (mm^2^)	40,186.98 ± 21,796.96	45,479.55 ± 57,223.05	0.554
Volume (cm^3^)	124.97 ± 244.24	78.42 ± 64.22	0.454

**Table 4:** Univariate analysis of radiomics comparing delayed burr-hole trepanation and conservative.

Variable	Burr-hole (n = 16)	Conservative (n = 38)	*p*-Value
Elongation	2.03 ± 0.65	1.61 ± 0.45	0.008
Feret Diameter (mm)	137.57 ± 29.91	125.94 ± 26.68	0.164
Sphericity	0.34 ± 0.15	0.24 ± 0.07	0.004
Surface area (mm^2^)	45,479.55 ± 57,223.05	26,001.05 ± 21,127.12	0.072
Volume (cm^3^)	78.42 ± 64.22	48.19 ± 50.72	0.071

Elongation emerged as a statistically significant parameter in both group comparisons (*p* = 0.001 vs. craniotomy; *p* = 0.008 vs. conservative). In addition, sphericity was significantly higher in the burr-hole group compared with conservative management (*p* = 0.004).

All variables with *p* < 0.05 in univariable analysis were considered for entry into a multivariable binary logistic regression model using backward selection. Prior to model fitting, multicollinearity was assessed. Spearman correlation coefficients between predictors remained <0.2 and non-significant (all *p* > 0.09), and variance inflation factors were below 1.2 for all variables, excluding relevant collinearity. Accordingly, SOFA score, leukocyte count, and elongation were entered into the multivariable regression model. In the final model, only elongation emerged as an independent predictor of hematoma chronification amenable to safe burr-hole evacuation (OR 22.0, 95% CI 3.67–132.6; *p* = 0.001), whereas SOFA score and leukocyte count did not remain statistically significant (Table 5).

**Table 5:** Results of the binary logistic regression (backward LR) predicting burr-hole trepanation compared to primary craniotomy.

Variable	Odds Ratio	95% CI	*p*-Value	Wald Statistic
SOFA score	0.80	0.58-1.10	0.135	1.88
Leukocytes	0.88	0.72-1.06	0.176	1.82
Elongation	22.04	3.67-132.60	0.001	11.41

Although postoperative epilepsy reached statistical significance in univariable testing, it was not considered for multivariable regression modeling, as epilepsy represents a clinical manifestation of hematoma chronification rather than an independent predictor available at baseline.

**Fig. 2:**
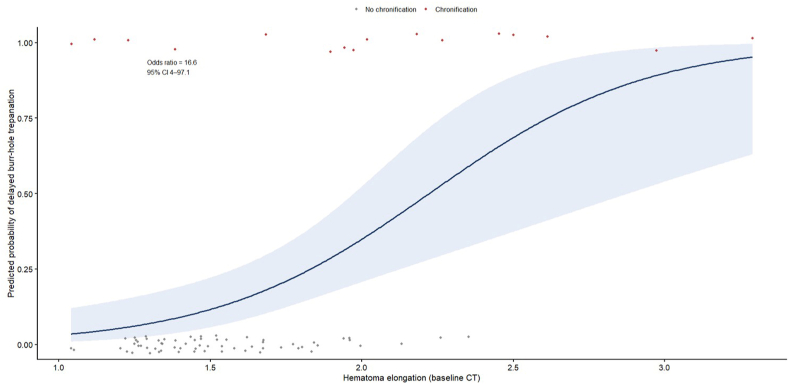
Association between hematoma elongation and risk of chronification. Logistic regression curve illustrating the predicted probability of delayed burr-hole trepanation according to hematoma elongation measured on admission CT. The solid line represents the fitted logistic model, and the shaded area indicates the 95% confidence interval. Individual data points are shown for patients without chronification (gray) and with chronification (red). Elongation was significantly associated with chronification (standard logistic regression OR 16.6; penalized Firth OR 10.7; p = 0.002).

Sensitivity analyses confirmed stability of the association. Standard logistic regression yielded an OR of 16.6 for elongation. Penalized (Firth) multivariable regression resulted in an OR of 10.73 (95% CI 2.15–78.16; p = 0.002). Bootstrap resampling (1000 iterations) demonstrated a stable positive effect, and leave-one-out analysis showed odds ratios ranging between 14.1 and 28.4 across iterations. Model discrimination was acceptable (AUC = 0.75; 95% CI 0.56–0.93). The association between elongation and predicted probability of chronification is illustrated in Fig. 2.

In addition to the comparison between burr-hole trepanation and primary craniotomy, a second regression model was calculated to contrast burr-hole trepanation with conservative management (Table 6). Prior to model fitting, multicollinearity was systematically assessed. Although variance inflation factors remained <1.2 for all predictors, Spearman correlation analysis revealed significant associations between roundness and both leukocyte count (r = −0.31, *p* = 0.021) as well as elongation (r = −0.40, *p* = 0.003). Given this overlap, roundness was excluded from the multivariable analysis to avoid redundancy.

**Table 6:** Results of the binary logistic regression (backward LR) predicting burr-hole trepanation compared to conservative.

Variable	Odds Ratio	95% CI	*p*-Value	Wald Statistic
Male sex	0.31	0.08 – 1.18	0.085	2.96
Leukocytes	0.87	0.72- 1.05	0.136	2.22
Elongation	4.02	1.24 – 13.09	0.021	5.36

## 5 Discussion

In this retrospective single-center study, we investigated clinical, laboratory, and radiomic predictors of hematoma progression in patients with traumatic aSDH. The key finding was that morphological radiomic features, particularly hematoma elongation, consistently emerged as independent predictors of subsequent delayed burr-hole trepanation, whereas hematoma volume was not associated with this outcome. Patients who eventually required burr-hole trepanation further exhibited a less severe clinical status, reflected by lower SOFA scores, and experienced fewer postoperative seizures compared with those undergoing primary craniotomy. These observations suggest that radiomic shape descriptors, rather than size, may provide critical information for identifying patients at risk of requiring delayed burr-hole intervention.

Taken together, these findings are consistent with and extend our previous work. The RADAR and PROMISE studies highlighted the prognostic relevance of radiomic morphology in aSDH, while the KEPPRA study demonstrated that radiomic features such as elongation may help predict postoperative epilepsy in patients undergoing craniotomy (Richter et al., 2025; Guranda et al., 2025a, 2025b). Building on this foundation, the present BRIDGE study shifts the focus from general outcome prediction toward stratifying patients who can safely undergo chronification and delayed burr-hole trepanation.

Management of aSDH remains particularly challenging in elderly patients, who constitute the majority of cases due to demographic aging (Evans et al., 2019) and widespread prescription of antithrombotic medication (Gaist et al., 2017). In this vulnerable population, even low-energy falls and minor head impacts frequently result in life-threatening aSDH (Lucke-Wold et al., 2016). While the standard of care remains urgent craniotomy in cases with mass effect, multiple studies have demonstrated poor outcomes in elderly cohorts, with mortality rates of 40–50% and unfavorable functional outcomes in up to 80% of cases (Manivannan et al., 2021). By contrast, cSDH is effectively treated with burr-hole trepanation, which is minimally invasive, widely accepted, and associated with markedly lower complication rates (Zumofen et al., 2009). Our findings suggest that a subset of aSDH patients may follow an “acute-to-chronic” pathway, ultimately becoming candidates for burr-hole evacuation rather than high-risk craniotomy.

This issue is particularly relevant in the German health care context. Octogenarians and nonagenarians, who represent an increasing proportion of patients with aSDH, and their families frequently decline acute craniotomy due to its high risks and limited functional recovery (Duehr et al., 2022).

In elderly patients, brain atrophy provides substantial compensatory capacity for space-occupying lesions (Ogashiwa et al., 1987). As a result, even large-volume aSDHs may remain clinically silent in this subgroup. This highlights the need for a radiomic marker that renders the process of chronification both feasible to detect and safe to anticipate. A three-dimensional analysis of hematoma shape may serve this purpose. Our findings support this hypothesis by showing that hematoma volume itself was not a significant predictor of delayed burr-hole trepanation.

However, many of these patients would accept burr-hole surgery if the hematoma evolves into a chronic form. Reliable prediction of chronification could therefore improve the indication process, allowing clinicians to avoid futile craniotomies, reduce perioperative morbidity, and respect patient-centered goals of care.

When contrasting delayed burr-hole trepanation with conservative management, our results further underline the clinical relevance of radiomic prediction. While some patients can be safely managed without surgery, others will eventually require burr-hole evacuation. Identifying these individuals at baseline is crucial, as it may justify closer clinical surveillance and tailored follow-up regimens. Radiomic shape features, particularly elongation, may therefore support risk stratification between conservative management and secondary burr-hole intervention.

Radiomic parameters derived from admission imaging are not intended to replace established clinical and radiological criteria for urgent craniotomy. Rather, elongation may serve as an adjunctive risk stratification marker in neurologically stable patients, supporting individualized follow-up and surveillance strategies. Decisions regarding acute surgical intervention remain primarily guided by neurological status, mass effect, and dynamic clinical evolution.

An additional finding of our study was the consistently lower leukocyte count in patients who later required burr-hole trepanation compared with both craniotomy and conservative cohorts. Although leukocytes did not retain significance in multivariable analysis, their univariable association raises interesting pathophysiological considerations. One possible explanation is that elevated leukocyte counts may reflect a more pronounced systemic inflammatory response to traumatic brain injury. Experimental and clinical data indicate that circulating neutrophils mount an oxidative burst proportional to injury severity, which has been associated with secondary neurological deterioration (Liao et al., 2013; Di Battista et al., 2016). Conversely, lower leukocyte levels could indicate a less inflammatory milieu that favors hematoma persistence and gradual evolution into the chronic stage (Edlmann et al., 2017). These hypotheses warrant further validation in larger, prospective studies integrating systemic inflammatory markers with radiomic features.

**Fig. 3:**
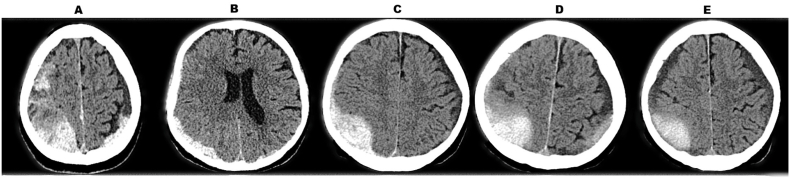
Representative evolution from acute to chronic subdural hematoma under conservative management. Serial axial non-contrast CT scans of a patient with traumatic acute subdural hematoma under therapeutic anticoagulation (phenprocoumon) and left ventricular assist device (LVAD) support. (A) Admission CT demonstrating acute hyperdense subdural hematoma with localized mass effect but preserved midline alignment and no focal neurological deficit. (B) Early follow-up imaging showing persistent mass effect without clinical deterioration. (C) Day 3 CT illustrating beginning density changes consistent with evolving hematoma organization. (D) Day 7 CT demonstrating progressive internal heterogeneity and decreasing acute hyperdensity. (E) Day 14 CT showing further hypodensification and radiological features consistent with chronification.

Importantly, early identification of patients prone to chronification is not solely relevant for treatment selection but also for anticipating delayed clinical deterioration. Even patients who initially present in a stable neurological condition may develop progressive membrane formation and gradual hematoma expansion during the subacute phase. This process can result in increasing mass effect and midline shift, occasionally leading to sudden neurological decline after an initially uneventful course. Without prior risk stratification, such deterioration may occur unexpectedly and require urgent intervention under less controlled circumstances. In this context, radiomic morphology may provide an additional tool to guide structured follow-up imaging and closer surveillance in patients at higher risk (see Fig. 3 for a representative example of subacute evolution).

Lower SOFA scores in the burr-hole group may indicate reduced systemic organ dysfunction at presentation, translating into greater physiological reserve and clinical stability. Such patients may tolerate the hematoma without immediate deterioration, allowing progression to the chronic stage. In contrast, higher SOFA scores likely reflect a more fragile systemic state that accelerates acute decompensation and necessitates early craniotomy. Although SOFA did not remain significant in multivariable analysis, its consistent association suggests that systemic status contributes to the clinical trajectory of aSDH.

Based on our results, we propose that radiomic shape features, particularly elongation, may be incorporated into clinical algorithms for aSDH management. In patients with relatively mild clinical status and imaging patterns suggestive of chronification, a strategy of initial observation with the option of delayed burr-hole trepanation may be justified. Conversely, patients with severe neurological impairment or radiological signs of mass effect remain candidates for urgent craniotomy. Importantly, our findings do not advocate withholding life-saving surgery in deteriorating patients but highlight the potential to individualize decision-making in stable patients at high surgical risk.

## 6 Limitations

This study has several limitations. First, its retrospective, single-center design and the small size of the chronification group restrict the generalizability of our findings. Nevertheless, the consistent association of elongation with hematoma chronification across both analyses supports its validity as a potential radiomic parameter. Second, hematoma segmentation was performed in a semi-manual workflow which, despite standardization and consensus review, introduces observer-dependent variability and limits scalability. Independent dual segmentation masks were not prospectively archived, precluding formal calculation of interobserver agreement metrics such as intraclass correlation coefficients. Third, the wide confidence interval observed for elongation in the multivariable analysis (95% CI 3.67–132.6) reflects limited precision of effect estimation and should be interpreted with caution, particularly given the small chronification subgroup. Finally, long-term follow-up data were not systematically available, precluding conclusions on functional outcome beyond discharge. Future research should address these limitations through prospective, multicenter designs, automated radiomic pipelines, and extended follow-up to confirm the clinical utility of elongation and other morphological features for individualized risk prediction.

## 7 Conclusion

The BRIDGE study demonstrates that hematoma chronification is not determined by hematoma volume but rather by morphological radiomic features, with elongation emerging as the most consistent predictor. Such patients exhibit less severe clinical profiles and fewer epileptic seizures, highlighting the clinical relevance of individualized treatment allocation. Integrating radiomic parameters into decision-making may help identify patients who can safely avoid acute craniotomy and benefit instead from delayed burr-hole trepanation, thereby improving both outcomes and alignment with patient preferences.

## Authors contributions

AG and MV contributed to the conception and design of the study.

AG and MV were responsible for data acquisition and analysis.

AG, JW, SL, FA, AEB, TW contributed to the writing of the manuscript.

EG and MW provided supervision and critical revision of the manuscript.

## Ethic approval

This study was conducted in accordance with the principles of the Declaration of Helsinki. Ethical approval was obtained from the Ethics Committee of the University of Leipzig (protocol number 362/23-ek; date of approval: 21 November 2023).

## Funding

This publication was supported by the Open Access Publishing Fund of Leipzig University.

## Declaration of competing interest

The authors declare that they have no known competing financial interests or personal relationships that could have appeared to influence the work reported in this paper.

## Data Availability

The data used and/or analyzed in the current study are available from the corresponding author upon reasonable request (AG).
